# A Stroke Risk Detection: Improving Hybrid Feature Selection Method

**DOI:** 10.2196/12437

**Published:** 2019-04-02

**Authors:** Yonglai Zhang, Yaojian Zhou, Dongsong Zhang, Wenai Song

**Affiliations:** 1 Medical Big Data Institute Software School North University of China Taiyuan China; 2 Department of Business Information Systems and Operations Research Belk School of Business University of North Carolina Charlotte, NC United States

**Keywords:** machine learning, stroke, risk, feature selection, WRHFS

## Abstract

**Background:**

Stroke is one of the most common diseases that cause mortality. Detecting the risk of stroke for individuals is critical yet challenging because of a large number of risk factors for stroke.

**Objective:**

This study aimed to address the limitation of ineffective feature selection in existing research on stroke risk detection. We have proposed a new feature selection method called weighting- and ranking-based hybrid feature selection (WRHFS) to select important risk factors for detecting ischemic stroke.

**Methods:**

WRHFS integrates the strengths of various filter algorithms by following the principle of a wrapper approach. We employed a variety of filter-based feature selection models as the candidate set, including standard deviation, Pearson correlation coefficient, Fisher score, information gain, Relief algorithm, and chi-square test and used sensitivity, specificity, accuracy, and Youden index as performance metrics to evaluate the proposed method.

**Results:**

This study chose 792 samples from the electronic records of 13,421 patients in a community hospital. Each sample included 28 features (24 blood test features and 4 demographic features). The results of evaluation showed that the proposed method selected 9 important features out of the original 28 features and significantly outperformed baseline methods. Their cumulative contribution was 0.51. The WRHFS method achieved a sensitivity of 82.7% (329/398), specificity of 80.4% (317/394), classification accuracy of 81.5% (645/792), and Youden index of 0.63 using only the top 9 features. We have also presented a chart for visualizing the risk of having ischemic strokes.

**Conclusions:**

This study has proposed, developed, and evaluated a new feature selection method for identifying the most important features for building effective and parsimonious models for stroke risk detection. The findings of this research provide several novel research contributions and practical implications.

## Introduction

### Background and Research Objective

Stroke is the second most popular cardiovascular disease (CVD). The World Health Organization estimated that 17.7 million people died from CVDs in 2017, of which 6.7 million had stroke, representing 31% of all deaths caused by diseases in the world [1]. The epidemiological characteristics of stroke in developing countries have gradually become closer to those of developed countries [2]. The prevalence and mortality of stroke are still on the rise. As of 2016, there were 13 million people with stroke in China [3]. Stroke prevention was the theme set by the World Stroke Organization for the 2017 World Stroke Day. Therefore, timely detection and prevention of stroke become essential.

People may go to a hospital for a full physical examination to assess stroke risk. Specific examination items include blood biochemical tests, blood pressure, electrocardiogram, vascular ultrasound, vascular computerized tomography angiography, magnetic resonance angiography, electroencephalography, magneto encephalography, single photon emission computerized tomography, positron emission computerized tomography, magnetic resonance imaging, and digital subtraction angiography. Plaque image analysis based on image segmentation technology has also been explored for risk detection of strokes [4,5].

As traditional medical risk assessment is expensive and not scalable, automated detection of stroke risk has been increasingly studied in recent years (eg, [6-11]), which falls into 2 broad categories: stroke risk assessment modeling and brain image analysis. Many countries have employed automated detection models for stroke, such as systematic coronary risk evaluation [12], QRISK (QRFSEARCH cardiovascular risk algorithm) [13], and Reynolds risk score [14]. The Framingham risk assessment model is a typical risk detection model of stroke. Pencina et al used an extended Framingham model to develop a 30-year risk detection model with data collected from 4506 patients aged 20 to 59 years [15]. The model detected the 30-year risk using 8 risk factors, including gender, antihypertensives, blood pressure, total cholesterol, high-density lipoprotein (HDL), smoking, impaired glucose tolerance, and left ventricular hypertrophy. Flueckiger et al further extended the Framingham model to establish a score detection model of stroke in a multiethnic study of atherosclerosis in conjunction with nontraditional risk markers [16]. The detection model included demographics, medical history, anthropometrics, and conventional risk factors. However, the Framingham model overestimates the risk of stroke in China because of obvious differences in the disease spectrum and risk factors [17,18]. A joint Chinese-American research group constructed a risk detection model of ischemic stroke and hemorrhagic stroke with 6 risk factors, including systolic blood pressure, sex, age, total cholesterol (TC), diabetes, and smoking [19].

Stroke consists of ischemic stroke and hemorrhagic stroke. Ischemic stroke accounts for 60% to 80% of stroke occurrence in China, which is the main context of this study. The detection of risk for ischemic stroke is aimed to reduce or prevent the incidence of clinical events and premature death associated with ischemic stroke by early prevention. A key limitation of existing research on stroke risk assessment lies in the lack of systematic guidance for feature selection while building stroke risk detection models, which is essential to the performance of such models. Previous studies chose predictive features largely in an ad hoc manner and did not incorporate the latest results of medical research. So, the core research question of this study is how to select important risk factors that should be included in a risk detection model for ischemic stroke as predictive features?

To address this research question, we proposed, developed, and evaluated a new hybrid feature selection method, namely weighting- and ranking-based hybrid feature selection (WRHFS). WRHFS integrates the strengths of various filter algorithms and deploys continuous weighting and ranking of individual features by following the principle of a wrapper approach. It then selects the top N ranked features as the most important features. This study makes a significant research contribution by proposing a new methodological approach to feature selection, which can lead to improved performance of risk detection models.

### Related Work

The key to accurate stroke risk detection is to select the most important and influential features of stroke patients, which may vary among patients at different regions.

Past research has shown that stroke is significantly associated with age [20], gender [21], blood pressure [20,21], low-density lipoprotein [22], triglyceride [23], drinking [24], smoking [25], creatine kinase (CK) [25], height [26], TC [27], HDL [24,27], body mass index (BMI) [22,25,28], serum total cholesterol [22,29], smoking [22,24,30], and diabetes [22,31]. Recently, some new risk factors have been discovered by medical research. For example, alkaline phosphatase [32] and hypercholesterolemia [33] are found to increase the probability of the mortality of stroke patients. Studies have also shown that there is a clear epidemiological relationship between stroke risk and hyperlipidemia [34]. However, no single study has used all features that are theoretically related to stroke because of their availability in data.

Traditionally, detectors of stroke risk were identified based on the findings of medical research and practice. However, collecting data for risk factors (also referred to as features in this paper) based on the results of medical research is extremely difficult. In the past decade, there has been increasing research on building automated stroke risk detection models by leveraging machine-learning techniques and patient data. One of the essential steps in building such models is to select effective features (ie, influential factors) that are associated with stroke, which is often referred to as the feature selection process. We categorized feature selection methods used in automated stroke risk detection models into semisupervised, unsupervised, and supervised methods [35,36], as summarized in Table 1. Semisupervised feature selection methods are suitable for datasets with a small number of labeled samples and a large number of unlabeled samples [37]. The key challenge lies in how to use the labeled samples to efficiently process the unlabeled samples. At present, unsupervised feature selection methods mainly focus on clustering-based models, for example, Laplacian score [38], trace ratio [39], and sparsity regularization–based models [40]. For example, a coregularized unsupervised feature selection algorithm was proposed in a study by Zhu et al [41], which was intended to ensure that the selected features could preserve both data distribution and reconstruction.

**Table 1:** Classification of feature selection methods.

Methods		Rationale	Limitations	Sample studies	
**Supervised**				
	Filter	Mutual information based	Signal objective function	[42]
		Ranking based	Neglecting the correlation between the features and class labels	[43]
		Weighting based	Lacking the uniform standards of selecting features	[44]
	Wrapper	Evaluating the accuracy of the classifier	Overfitting and high computational complexity	[45]
	Hybrid	Guiding the wrapper using a filter	Only for certain specific fields	[46]
Semisupervised		Guiding by the labeled samples	Relying on small labeled samples	[37]
Unsupervised		Clustering-based models	Relying on certain data distribution	[40]

Supervised feature selection methods can be further divided into filter, wrapper, and hybrid methods. The filter feature selection method consists of mutual information and ranking- and weighting-based methods. Mutual information–based filter methods use mutual information to evaluate the relevance of features to class labels and the redundancy of candidate features. However, they suffer from the problem that the objective function only uses a single statistic measure of a dataset (eg, standard deviation, information gain [42,47], or Fisher score [48]), while ignoring the fusion of multiple measures. For example, a standard deviation–based filter model relies on the distance between feature value and mean value for feature selection. Information entropy is often used to measure the uncertainty of the value of a random variable. Information gain, referred to as the change in information entropy, of a feature in a dataset can be used to rank features. The greater the information gain is, the more a feature contributes to classification.

Feature ranking methods (eg, maximal relevance and minimal redundancy objective [43]) are independent of classification algorithms. They select a feature subset with metrics such as the Relief algorithm [49-51] and correlation estimate [43,52]. The Relief algorithm has been successfully applied to feature weighting because of its simplicity and effectiveness [41,42,47]. It is inspired by instance-based learning algorithms according to their ability to discriminate neighboring patterns. Linear in-time complexity, Relief has a great advantage in computational efficiency. It selects a sample x randomly and then finds the nearest neighbor sample NearHit(x) in the same class and the nearest neighbor sample NearMiss(x) in another class. However, its significant disadvantage lies in that feature ranking overemphasizes the relevance of a certain feature to a class label or the correlation with other individual features based on a single objective function, while neglecting the correlation between the combined features and a class label. In addition, when the independent relevance of a feature is emphasized, the redundancy of feature ranking will be increased, which contradicts to the objective of minimization of redundancy.

Feature weighting methods attempt to assign a weight value, usually in the range of 0 to 1, to each feature. Features with weights near 1 will be selected to form a feature set, whereas other features will be discarded [44]. Those methods are lacking the uniform standards for selecting features because of the fuzziness of *near 1*. Overall, filter models select features by weighting and ranking features based on their statistical relevance to class labels and a threshold to filter out irrelevant features to improve the classification accuracy [53].

Wrapper methods search for the optimal subset of features in a feature space and use a classifier to evaluate the effectiveness of a feature subset. For a particular classifier, wrapper methods may find good feature subsets [45]. However, they are prone to overfitting and high computational complexity.

Hybrid models use a filter model to guide a wrapper model to solve these problems of filter and wrapper methods [46,54-56]. In summary, for stroke risk detection, traditional feature selection methods have a variety of limitations, negatively affecting the quality of selected features and the performance of stroke risk detection models.

## Methods

### Design

**Figure 1:**
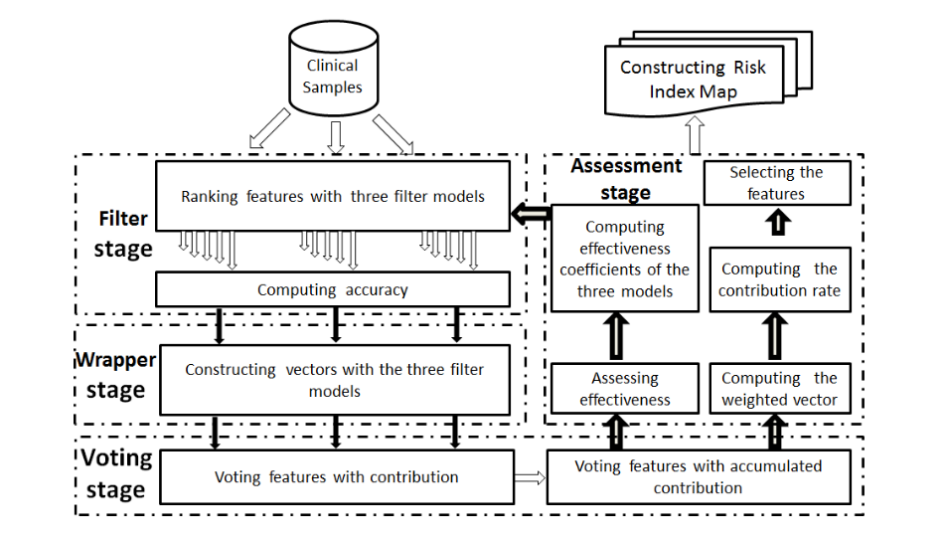
Weighting- and ranking-based hybrid feature selection.

In this study, we proposed a new hybrid feature selection model called WRHFS, which selects features by integrating various filter and wrapper methods. Being different from previous hybrid methods, WRHFS selects the best n filter models (in this study, n=3) from a candidate set to guide a wrapper model. Figure 1 shows the process of WRHFS, which consists of 4 parts. WRHFS selects the top 3 filter methods from a set of candidate filter models.

- Filter stage: ranking features with multiple filter models.

(1) Randomly choosing 3 different models from a set of candidate filter models.

(2) Ranking features based on each filter model. WRHFS uses the ordered features of the filter models to train multiple classification models based on the backward searching strategy and measures classification accuracies and contribution vectors ω from the 3 filter models.

- Wrapper stage: constructing an aggregated contribution vector W using the 3 contributions of individual features from the 3 filter models.

(3) Creating W by aggregating the 3 contribution vectors ω_i_ (i=1, 2, and 3) generated by the 3 filter models. W is expressed as follows:

W=∑ω
_i_, i=1, 2, 3 (1)

- Voting stage: voting features based on a contribution matrix.

(4) Building a classification contribution matrix C based on the 3 contribution vectors. C is expressed as follows:

C=[ω1ω2ω3] (2)

(5) Building a cumulative classification contribution matrix D on the 3 contribution vectors ω_i_. D_1_, D_2,_ and D_3_ are the cumulative contribution vectors based on the vectors ω_i_, respectively. D is expressed as follows:

D=[D
_1_D
_2_D
_3_] (3)

- Assessment stage: assessing the effectiveness of the 3 filter feature selection models and selecting the most important features.

(6) Building the effectiveness coefficient vector P of the 3 models and assessing the effectiveness of those models. P is a 3-dimensional vector, which contains the effectiveness coefficients of the 3 filter models. P is defined as follows:

P=W x D (4)

WRHFS assesses the first 3 filter models according to the effectiveness coefficient vector P, then replaces the worst filter model by choosing a filter model from the remaining filter models in the candidate set. Then, it repeats steps (1) to (6) until the candidate model set is empty. Finally, the top 3 filter models will be chosen based on the effectiveness coefficient vector P to develop the optimal feature selection model.

(7) Calculating the weight W_r_ of each selected individual feature. The formula of W_r_ is as follows:

W
_r_= P x C
^T^(5)

(8) Ranking features based on the weights W_r_.

(9) Selecting the top N key features based on their weights, in which the cumulative contribution of key features is more than 50% and generating the risk index map for diseases using the surface fitting technique based on key features.

### Performance Measures

We evaluated the performance of WRHFS in terms of sensitivity, specificity, accuracy, and Youden index using a real-world dataset. We adopted the most common performance measures of classification models in medical diagnostics, including sensitivity, specificity, accuracy, and Youden index. There are 4 categories of potential outcomes: true positive (people with ischemic risk correctly identified), false positive (healthy people incorrectly identified as having risk), true negative (healthy people correctly identified as healthy), and false negative (people with ischemic stroke incorrectly identified as without risk). Sensitivity (also called the true positive rate or recall) measures the proportion of actual positives correctly detected as people with stroke risk, as shown in equation (6). Specificity (also called the true negative rate) measures the proportion of actual negatives that are correctly identified as healthy people, as shown in equation (7). Accuracy is defined as equation (8). Among the 3 measures, sensitivity is the most important medical criterion. Youden index, also called Youden J statistic, captures the performance of a dichotomous diagnostic test. Youden index is defined in equation (9).

Sensitivity = True positives / (True positives + False negatives) = True positives / Sick individuals (6)

Specificity = True negatives / (True negatives + False positives) = True negatives / Well individuals (7)

Accuracy = (True positives + True negatives) / All = True individuals / All individuals (8)

Youden index=Sensitivity + Specificity – 1 (9)

We employed 6 filter methods commonly used in the medical field, including those based on standard deviation [57], Pearson correlation coefficient [58], Fisher score [59], information gain [60], Relief [61], and chi-squared test [62]. We used a 10-fold cross-validation to train and test classification models. In the evaluation, we selected methods based on standard deviation, Pearson correlation coefficient, and Fisher score, initially. We adopted support vector machine (SVM), Bayes [63], classification based on associations [64], back-propagation neural networks [65], classification and regression tree [66], C4.5 (the decision tree learner) [67], and extreme learning machine [68] to build different detection models because they are the commonly used classification algorithms. Afterward, we kept the top 3 filter methods as the benchmark feature selection models and compared their performances against that of the proposed WRHFS method.

## Results

### Dataset

This study adopted a retrospective cohort. We collected a dataset that consisted of records of 80,672 patients from a community hospital. Among them, 13,421 patients suffered from ischemic stroke in the past 5 years. We extracted their records before their diagnoses of ischemic stroke. Given the purpose of modeling, we only chose and used features that did not have missing values in the entire dataset. We did not use missing value supplementation techniques because of concerns of possible biases or noises that may incur when applying those techniques.

At the end, there were 792 complete records in the dataset, each including 24 blood test features, as shown in Table 2. We also included 4 demographic features of the patients, including gender, age, height, and BMI. Descriptive statistics of age and gender are reported in Table 3. Among the 792 qualified patient records, 398 were diagnosed with ischemic stroke and labeled as class 1 instances, whereas the remaining 394 were not diagnosed with ischemic stroke and were labeled as class 2 instances.

### Weighting of Features Using Weighting- and Ranking-Based Hybrid Feature Selection

WRHFS assessed the effectiveness of filter feature selection methods given in the dataset. Afterward, we discarded the filter method with lower effectiveness coefficients. The greater the coefficient, the higher the effectiveness. As shown in Table 4, information gain, Relief, and standard deviation led to the top 3 model performances. Table 5 shows the weights of the features based on standard deviation The weights of the features based on Relief and information gain are shown in Multimedia Appendices 1 and 2. Here, “accuracy” refers to the classification accuracy of the SVM classifier on the basis of the backward searching strategy, whereas “C” and “q” indicate the optimal penalty parameter and the kernel bandwidth in the SVM algorithm, respectively. Feature contribution is reflected by the difference between the accuracy of a model including a specific feature versus the accuracy of the model without it. We used the normalized result between 0 and 1 to eliminate the difference between positive and negative. “SD (0-1)” and “Contribution (0-1)” indicate the normalized results of “accuracy” and “contribution”, respectively. “Weight” reflects the overall performance of the features, which is the sum of standard deviation (0-1) and the contribution (0-1).

In Table 6, “weight sum” indicates the sum of the weights calculated by the 3 filter feature selection models. In Table 7, columns 2 to 4 compose the contribution matrix C, whereas columns 5 to 7 compose the cumulative contribution matrix D. In Table 8, the results of the weighted sum of the features using WRHFS are sorted in decreasing order, with a larger weight indicative of higher importance.

Table 9 presents the optimal performance of the trained risk detection models in terms of the 4 measures, including sensitivity (the positive detection rate), specificity (the negative detection rate), accuracy (the overall classification accuracy), and Youden index. From Table 9 it can be seen that the proposed WRHFS method achieved sensitivity of 82.7% (329/398) and classification accuracy of 81.5% (645/792) using only the top 9 features, and different classification models achieved the best performance when using different features. For example, information gain achieved the best classification accuracy of 72.5% (574/792) when using the top 10 features, and the accuracy began to decline when adding the eleventh feature. Similarly, standard deviation achieved the best classification accuracy of 73.2% (580/792) with the top 20 features presented in Table 5, and Relief achieved 72.9% (577/792) with the top 13 features. Therefore, we calculated sensitivity, specificity, accuracy, and Youden index of those methods by only using those optimal features that resulted in the best performed models. Among these feature selection methods, the proposed WRHFS method resulted in the highest performance measures with the fewest features. As shown in Table 8, Age, α-HBD, SCr, LDH, Height, TBIL, CK, Apo-B, and CK-MB are the top 9 most important features among the 28 features identified by WRHFS. Their cumulative contribution was 0.51. Table 10 presents the performances of models developed by different classifiers, as explained in Section “Performance Measures” using the same 9 features identified by WRHFS. Among all the models, SVM using WRHFS achieved the best performance in all 4 measures.

**Table 2:** 24 blood test items.

Full name	Abbreviation	Unit	Type of data
α Hydroxybutyric dehydrogenase	α- HBD	IU/L	Integer
Gamma glutamyl transpeptidase	GGP	IU/L	Integer
Lactate dehydrogenase	LDH	mmol/L	Real
Low-density lipoprotein	LDL	mmol/L	Real
High-density lipoprotein	HDL	mmol/L	Real
Blood urea nitrogen	BUN	mmol/L	Real
Uric acid	UA	umol/L	Integer
Total cholesterol	TC	mmol/L	Real
Total bilirubin	TBIL	umol/L	Real
Total protein	TP	g/L	Integer
Triglyceride	TG	mmol/L	Real
Albumin	Alb	g/L	Integer
Direct bilirubin	DBIL	umol/L	Real
Alkaline phosphatase	ALP	IU/L	Integer
Serum phosphorus	PI	mmol/L	Real
Serum creatinine	SCr	umol/L	Integer
Creatine kinase	CK	IU/L	Integer
Creatine kinase isoenzyme	CK-MB	IU/L	Integer
Glucose	Glu	mmol/L	Real
Alanine aminotransferase	ALT	IU/L	Integer
Aspartate aminotransferase	AST	IU/L	Integer
Apolipoprotein A1	Apo-A1	g/L	Real
Apolipoprotein B	Apo-B	g/L	Real
Serum calcium	Ca	mmol/L	Real

**Table 3:** Descriptive statistics of age and gender of patients in the dataset (N=792)

Age (years) and gender		Statistics, n (%)
**≥ 45 and ≤60**		
	Male	105 (13.3)
	Female	167 (21.1)
**>60 and ≤75**		
	Male	151 (19.1)
	Female	246 (31.1)
**>75 and ≤90**		
	Male	76 (9.6)
	Female	47 (5.9)

**Table 4:** Effectiveness coefficients of the filter feature selection methods.

Method	Effective coefficient
Information gain	63
Relief	61
Standard deviation	52
Pearson correlation coefficient	49
Fisher score	46
Chi-squared test	40

**Table 5:** Weighting of the 28 features based on standard deviation.

Feature^a^	Standard deviation	C	q	Accuracy (%)	Contribution	SD (0-1)	Contribution (0-1)	Weight
CK	0.21	16	0.5	56.1	—^b^	1.00	1.00	—
LDH	0.21	64	0.5	57.1	1.00	0.99	0.30	1.00
α-HBD	0.19	128	8.0	58.6	1.52	0.91	0.37	1.52
Height	0.17	8	8.0	57.3	−1.26	0.81	0.00	−1.26
ALP	0.15	2	4.0	58.8	1.52	0.72	0.37	1.52
UA	0.10	1	8.0	58.6	−0.25	0.48	0.13	−0.25
SCr	0.09	16	4.0	61.9	3.28	0.41	0.60	3.28
GGP	0.08	16	2.0	61.6	−0.25	0.40	0.13	−0.25
TP	0.08	2	8.0	61.6	0.00	0.37	0.17	0.00
AGE	0.08	64	1.0	67.9	6.31	0.36	1.00	6.31
ALT	0.07	128	1.0	67.6	−0.38	0.31	0.12	−0.38
AST	0.06	64	1.0	67.9	0.38	0.27	0.22	0.38
CK-MB	0.05	128	0.2	69.2	1.26	0.23	0.33	1.26
Alb	0.05	128	0.1	69.6	0.38	0.22	0.22	0.38
TBIL	0.04	256	0.5	72.1	2.53	0.16	0.50	2.53
BMI	0.03	64	0.3	72.7	0.63	0.12	0.25	0.63
Glu	0.01	128	0.3	72.7	0.00	0.04	0.17	0.00
DBIL	0.01	64	0.5	73.1	0.38	0.04	0.22	0.38
BUN	0.01	64	0.5	73.0	−0.13	0.03	0.15	−0.13
TC	0.01	64	0.5	73.2	0.25	0.02	0.20	0.25
LDL	0.01	128	1.0	73.0	−0.25	0.02	0.13	−0.25
TG	0.00	128	1.0	72.9	−0.13	0.02	0.15	−0.13
Gender	0.00	128	1.0	73.0	0.13	0.00	0.18	0.13
Ca	0.00	64	0.5	73.0	0.00	0.00	0.17	0.00
Apo-A1	0.00	128	1.0	73.2	0.25	0.00	0.20	0.25
HDL	0.00	128	1.0	73.1	−0.13	0.00	0.15	−0.13
Apo-B	0.00	128	1.0	73.1	0.00	0.00	0.17	0.00
PI	0.00	128	1.0	73.0	−0.13	0.00	0.15	−0.13

^a^The full forms of all abbreviations are shown in Table 2.

**Table 6:** Weighting of the 3 feature selection models.

Order	Feature^a^	Standard deviation	Relief	Information gain	Weight sum
1	α-HBD	0.9123	1.0000	0.0001	1.9124
2	GGP	0.4000	0.0657	0.0498	0.5156
3	Alb	0.2198	0.0592	0.0211	0.3001
4	LDL	0.0197	0.0026	0.0236	0.0459
5	TG	0.0156	0.0002	0.0001	0.0159
6	HDL	0.0032	0.0000	0.0010	0.0042
7	ALT	0.3120	0.0055	0.1141	0.4316
8	AST	0.2734	0.0366	0.0985	0.4085
9	SCr	0.4142	0.0637	0.0638	0.5417
10	CK	1.0000	0.5919	0.0549	1.6468
11	CK-MB	0.2303	0.0190	0.1657	0.4150
12	ALP	0.7239	0.0509	0.1051	0.8799
13	AGE	0.3574	0.0503	1.0000	1.4077
14	BUN	0.0296	0.0005	0.0845	0.1146
15	UA	0.4817	0.0024	0.0037	0.4878
16	LDH	0.9884	0.9582	0.0788	2.0254
17	Height	0.8145	0.4240	0.1235	1.3621
18	BMI	0.1171	0.0011	0.2146	0.3328
19	Gender	0.0049	0.0000	0.1349	0.1398
20	Ca	0.0040	0.0000	0.0000	0.0040
21	PI	0.0000	0.0001	0.0812	0.0813
22	Glu	0.0430	0.0009	0.4154	0.4593
23	Apo-A1	0.0036	0.0001	0.4525	0.4562
24	Apo-B	0.0013	0.0001	0.6987	0.7000
25	DBIL	0.0364	0.0003	0.2629	0.2996
26	TC	0.0248	0.0000	0.0382	0.0630
27	TBIL	0.1633	0.0323	0.5188	0.7143
28	TP	0.3667	0.0946	0.0417	0.5029

^a^The full forms of all abbreviations are shown in Table 2.

**Table 7:** Contribution of individual features.

Feature^a^	Contribution			Cumulative contribution		
	Standard deviation	Relief	Information gain	Standard deviation	Relief	Information gain
α-HBD	0.9123	1.0000	0.0001	1.6654	1.0000	6.2282
GGP	0.4000	0.0657	0.0498	2.8987	2.5000	5.1229
Alb	0.2198	0.0592	0.0211	4.9488	3.4428	5.8159
LDL	0.0197	0.0026	0.0236	6.5655	6.2714	5.5703
TG	0.0156	0.0002	0.0001	6.7155	7.8856	6.3685
HDL	0.0032	0.0000	0.0010	7.4155	9.4571	6.0966
ALT	0.3120	0.0055	0.1141	4.1821	6.0714	3.7369
AST	0.2734	0.0366	0.0985	4.3988	5.0000	4.0439
SCr	0.4142	0.0637	0.0638	2.7654	3.0857	4.8422
CK	1.0000	0.5919	0.0549	1.0000	1.6571	4.9562
CK-MB	0.2303	0.0190	0.1657	4.7321	5.8428	2.4474
ALP	0.7239	0.0509	0.1051	2.0320	3.6428	3.8158
AGE	0.3574	0.0503	1.0000	4.0654	4.6428	1.0000
BUN	0.0296	0.0005	0.0845	6.2321	7.3999	4.1930
UA	0.4817	0.0024	0.0037	2.1654	6.5428	5.9299
LDH	0.9884	0.9582	0.0788	1.2987	1.6571	4.5878
Height	0.8145	0.4240	0.1235	1.6654	1.7714	3.6492
BMI	0.1171	0.0011	0.2146	5.6988	6.8857	2.3070
Gender	0.0049	0.0000	0.1349	6.8988	9.2142	2.6492
Ca	0.0040	0.0000	0.0000	7.0655	9.7142	6.5264
PI	0.0000	0.0001	0.0812	7.7322	8.1285	4.3509
Glu	0.0430	0.0009	0.4154	5.8655	7.1428	2.0614
Apo-A1	0.0036	0.0001	0.4525	7.2655	8.4142	1.8246
Apo-B	0.0013	0.0001	0.6987	7.5822	8.7285	1.4386
DBIL	0.0364	0.0003	0.2629	6.0821	7.6285	2.3070
TC	0.0248	0.0000	0.0382	6.4322	8.9571	5.4387
TBIL	0.1633	0.0323	0.5188	5.4488	5.3857	1.7018
TP	0.3667	0.0946	0.0417	3.0654	2.0428	5.2808

^a^The full forms of all abbreviations are shown in Table 2.

**Table 8:** Weighting of the 28 features using weighting- and ranking-based hybrid feature selection.

Order	Feature^a^	Weight	Contribution	Cumulative contribution	Weight (0-1)
1	Age	176.31	0.13	0.13	1
2	α-HBD	88.36	0.06	0.19	0.42
3	SCr	83.02	0.06	0.25	0.38
4	LDH	70.59	0.05	0.30	0.30
5	Height	70.32	0.05	0.35	0.30
6	TBIL	66.18	0.05	0.39	0.27
7	CK	59.22	0.04	0.44	0.22
8	Apo-B	55.61	0.04	0.48	0.20
9	CK-MB	54.09	0.04	0.51	0.19
10	Alb	48.60	0.03	0.55	0.15
11	AST	47.49	0.03	0.58	0.15
12	GGP	45.36	0.03	0.61	0.13
13	DBIL	40.76	0.03	0.64	0.10
14	Glu	39.35	0.03	0.67	0.09
15	Gender	37.99	0.03	0.70	0.08
16	ALP	36.26	0.03	0.72	0.07
17	Apo-A1	35.60	0.03	0.75	0.07
18	TP	35.22	0.03	0.77	0.06
19	Ca	34.34	0.02	0.80	0.06
20	TC	34.34	0.02	0.82	0.06
21	BMI	33.90	0.02	0.85	0.06
22	HDL	33.16	0.02	0.87	0.05
23	BUN	32.92	0.02	0.89	0.05
24	PI	32.61	0.02	0.92	0.05
25	TG	32.37	0.02	0.94	0.05
26	UA	30.70	0.02	0.96	0.03
27	LDL	27.46	0.02	0.98	0.01
28	ALT	25.56	0.02	1.00	0

^a^The full forms of all abbreviations are shown in Table 2.

**Table 9:** Classification performances of support vector machine with different feature selection methods.

Method	Features	Sensitivity (N=398), n (%)	Specificity (N=394), n (%)	Accuracy (N=792), n (%)	Youden index
WRHFS^a^	9	329 (82.7)	317 (80.4)	645 (81.5)	0.63
Information gain	10	297 (74.6)	284 (72.1)	574 (72.5)	0.47
Relief	13	277 (69.6)	290 (73.7)	577 (72.9)	0.43
Standard deviation	20	283 (71.1)	291 (73.9)	580 (73.2)	0.45

^a^WRHFS: weighting- and ranking-based hybrid feature selection.

**Table 10:** Classification performances of different models with weighting- and ranking-based hybrid feature selection.

Classifier	Sensitivity (N=398), n (%)	Specificity (N=394), n (%)	Accuracy (N=792), n (%)	Youden index
SVM^a^	329 (82.7)	317 (80.4)	645 (81.5)	0.63
Bayes	319 (80.2)	197 (50.02)	520 (65.7)	0.30
CBA^b^	305 (76.6)	300 (76.1)	605 (76.4)	0.53
BPNN^c^	280 (70.4)	220 (55.8)	501 (63.2)	0.26
CART^d^	280 (70.4)	283 (71.8)	562 (71.0)	0.42
C4.5	269 (67.6)	302 (76.6)	571 (72.1)	0.44
ELM^e^	220 (55.3)	249 (63.2)	469 (59.2)	0.19

^a^SVM: support vector machine.

^b^CBA: classification based on associations.

^c^BPNN: back-propagation neural networks.

^d^CART: classification and regression tree.

^e^ELM: extreme learning machine.

**Figure 2:**
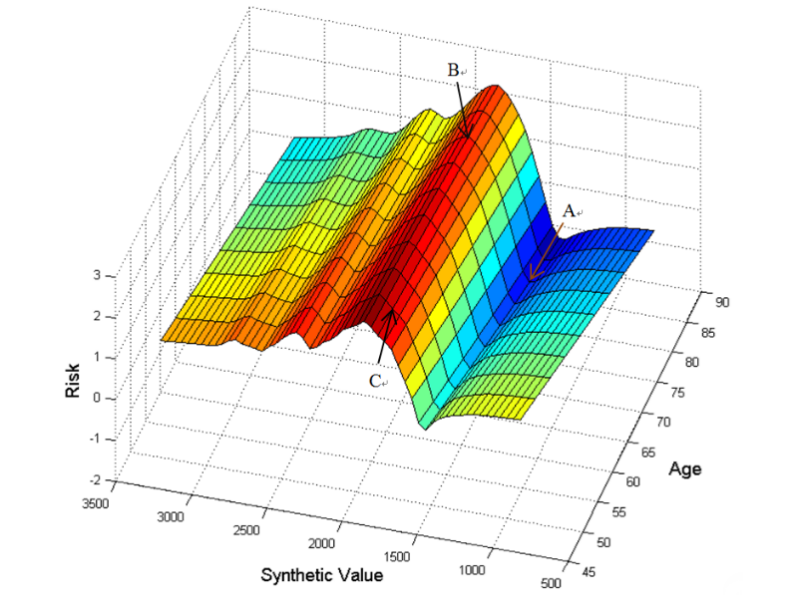
A surface chart for risk detection.

**Figure 3:**
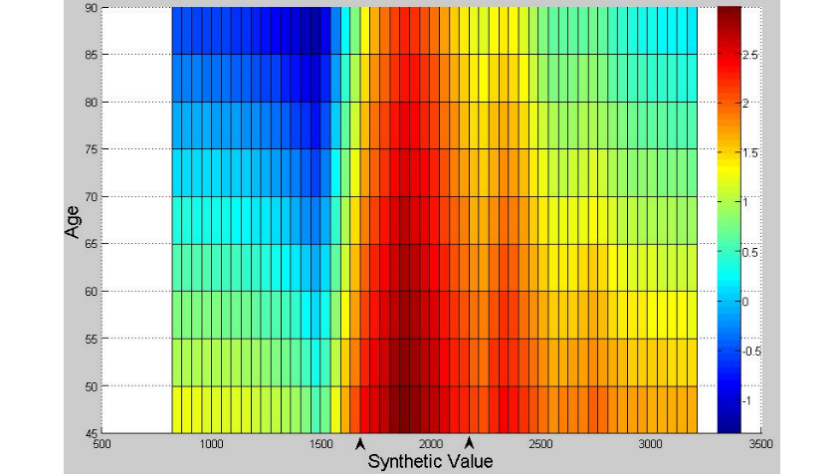
A risk index map for ischemic stroke detection.

We visualized the change trend of the risk levels of ischemic stroke in Figure 2 using the surface fitting technique based on the 9 key features. The synthetic value (SV) indicates the linear combination of the feature value and its weight. The risk of ischemic stroke is reflected in the SV, which is defined as follows:

SV=AGE+0.42 x α-HBD+0.38 x SCr+0.3 x LDH+0.3 x HEIGHT+0.27 x TBIL+0.22 x CK+0.2 x Apo-B+0.19 x CK-MB

where age, α-HBD, and other features indicate the feature values, and 0.42, 0.38, and other values are the weights associated with individual features. Figure 2 presents the surface chart for stroke risk detection, in which the Y axis represents the age between 45 and 90 years, the Z axis represents risk index of suffering from ischemic stroke, and the X axis represents the SV. Figure 3 presents the risk index map for ischemic stroke detection, which is a top view of Figure 2. There were 33 ranks of risk index: “≤1.5” means no risk; “>1.5 but ≤2” means low risk; and “>2” means high risk. Different colors indicate different levels of risks.

## Discussion

To address the limitations of existing risk detection models and expensive detection costs in hospitals, we proposed a new feature selection method, namely WRHFS, for risk detection of ischemic stroke. In this study, WRHFS selected features through the guidance of the top 3 filter methods based on the 28 risk factors. It provided an aggregated importance weight for each feature. As shown in Table 8, the top 9 features that achieved sensitivity of 82.7% (329/398) were selected for detecting the risk of ischemic stroke. WRHFS can also evaluate the effectiveness of the existing filter feature selection methods based on effective coefficients and choose the top 3 filter methods. On the basis of the sorted results of the importance weights of individual features, we chose 9 features and produced the change trend of risk levels and the risk index map for ischemic stroke.

### Principal Findings

We compared the performance of the proposed feature selection method WRHFS against those of standard deviation, Relief, and information gain. The results revealed that age is the most important influence factor because it has the largest weight value, which is consistent with the literature on stroke [6,13]. Through ranking the features in decreasing order of their importance to the performance of a risk detection model, WHRFS enables us to choose the most effective features that have the highest contributions to the performance measures of a model. Results of evaluation demonstrate that WRHFS can achieve a contribution rate of 0.51 with only the first 9 features, whereas the other 3 traditional feature selection methods require more. As a feature selection method, WRHFS is superior by being able to calculate effectiveness coefficients of individual features.

The contributions of the other 27 risk factors, excluding age, vary in the models constructed by the 4 feature selection methods, including WRHFS, standard deviation, Relief, and information gain. More specifically, the contribution of α-HBD assessed by Relief is significantly greater than that assessed by other feature selection methods, and the contribution of CK was ranked highest by standard deviation but almost 0 by Relief. It implies that a single objective function may not be able to measure the importance of risk factors comprehensively.

Age is the most important feature found in this study. The contribution of age to the model’s performance is approximately 13%. The risk of stroke was reflected in the SV. Therefore, age should be integrated in the SV. In general, the risk of ischemic stroke increases with age. As shown in Figure 2, A and B have the same age but are much older than C. However, B has higher risk than A because of the higher SV. In contrast, C is younger than A but has a higher risk than A also because of a higher SV. Therefore, the risk of ischemic stroke is influenced by the SV. A person would have low risk of ischemic stroke if the SV is far from the high-risk interval (HRI; ie, 1675, 2175), which is shown in Figure 3. The findings of this study will not only provide methodological guidance on how to select more effectiveness features for automated detection of stroke risk but also potentially help physicians improve their diagnosis in medical practice.

The major contribution of this research is WRHFS, a new generic feature selection method. WRHFS deploys continuous weighting and ranking of individual features by following the principle of a wrapper approach that integrates the strengths of various filter methods for feature selection. The evaluation shows that WRHFS can result in a superior risk detection model that achieves better performance with fewer features than the existing feature selection methods, demonstrating the effectiveness of WRHFS.

The findings of this study also provided multiple practical implications for physicians. First, the top 9 features are extremely easy to obtain. Physicians can calculate the corresponding SV and easily detect the ischemic stroke risk indexes using the risk index map as an auxiliary diagnostic method. As shown in Figure 3, the range between 1675 and 2175 of the SV (where the black arrow points) can be called the HRI. There seems a parabolic envelope curve. In addition, elderly people whose ages are between 70 and 90 years tend to have a high risk of ischemic stroke, whereas the risk becomes lower when the SV is smaller (800 to 1500) or larger (3000 to 3250). In addition, an automated stroke risk detection platform can be developed easily by use of the above findings for stroke during the physical examination of people.

### Limitations

This study has a couple of limitations that offer future research opportunities. First, the acquisition of medical samples is very difficult. We were unable to find data samples that included all of the risk factors that have been discovered in the literature. It would be worthy to conduct a future study with a larger and different dataset with more features to examine if the finding of this research can still hold. Second, we used a straightforward way to aggregate the rankings of individual filter methods, which may or may not be optimal. We plan to explore other means in future research.

### Conclusions

Automatic detection of stroke risks has been increasingly studied in recent years. How to select important factors for risk detection models is critical to the model’s performance. Existing research on automatic detection of stroke risks through machine learning faces a significant challenge in the selection of effective features as predictive cues. Therefore, how to develop more effective methods for feature selection is critical. This study proposed, developed, and evaluated a new feature selection method, which can help identify the most important features for building effective and parsimonious models for stroke risk detection. The proposed method, WRHFS, provides a novel methodological research contribution and practical implications.

## Appendix

Multimedia Appendix 1 Weighting of the 28 features based on Relief.

Multimedia Appendix 2 Weighting of the 28 features based on information gain.

## Abbreviations

BMI: body mass index
BPNN: back-propagation neural networks
CK: creatine kinase
CVD: cardiovascular disease
HDL: high-density lipoprotein
HRI: high-risk interval
SVM: support vector machine
SV: synthetic value
TC: total cholesterol
WRHFS: weighting- and ranking-based hybrid feature selection
