# Supplementary material for: A Stroke Risk Detection: Improving Hybrid Feature Selection Method
Source: J Med Internet Res. 2019 Apr 2;21(4):e12437. doi: 10.2196/12437 (PMC6466481; doi:10.2196/12437)
Supplement: Multimedia Appendix 1 [file jmir_v21i4e12437_app1.pdf]

Appendix 1: Weighting of the 28 Features Based on RELIEF

Feature	Relief	C	q	Accuracy (%)	Contribution	Relief (0-1)	Contribution (0-1)	Weight
α-HBD	1564.2	4	128	57.3	-	1.00	1.00	2.00
LDH	1498.8	0.5	256	60.9	3.54	0.96	0.66	1.62
CK	925.93	128	8	58.6	-2.27	0.59	0.00	0.59
Height	663.29	8	8	57.3	-1.26	0.42	0.11	0.54
TP	147.91	1	16	57.4	0.13	0.09	0.27	0.37
GGP	102.80	1	8	59.2	1.77	0.07	0.46	0.52
SCr	99.61	1	8	62.1	2.90	0.06	0.59	0.65
Alb	92.65	256	1	63.0	0.88	0.06	0.36	0.42
ALP	79.66	16	2	62.5	-0.51	0.05	0.20	0.25
AGE	78.76	128	1	69.1	6.57	0.05	1.00	1.05
AST	57.28	128	1	69.9	0.88	0.04	0.36	0.39
TBIL	50.51	64	0.5	71.1	1.14	0.03	0.39	0.42
CK-MB	29.69	256	0.25	72.9	1.77	0.02	0.46	0.48
ALT	8.59	128	0.5	72.6	-0.25	0.01	0.23	0.23
LDL	4.13	256	0.06	72.1	-0.51	0.00	0.20	0.20
UA	3.79	256	0.06	72.2	0.13	0.00	0.27	0.27
BMI	1.77	128	1	73.0	0.76	0.00	0.34	0.34
Glu	1.36	64	1	73.0	0.00	0.00	0.26	0.26
BUN	0.73	64	1	73.0	0.00	0.00	0.26	0.26
DBIL	0.54	128	1	72.7	-0.25	0.00	0.23	0.23
TG	0.38	128	1	72.7	0.00	0.00	0.26	0.26
PI	0.21	64	1	72.6	-0.13	0.00	0.24	0.24
Apo-A1	0.18	128	1	72.9	0.25	0.00	0.29	0.29
Apo-B	0.08	128	1	73.4	0.51	0.00	0.31	0.31
TC	0.06	128	1	73.1	-0.25	0.00	0.23	0.23
Gender	0.03	128	1	73.1	0.00	0.00	0.26	0.26
HDL	0.00	128	1	73.0	-0.13	0.00	0.24	0.24
Ca	0.00	128	1	73.0	0.00	0.00	0.26	0.26
