# Supplementary material for: A Stroke Risk Detection: Improving Hybrid Feature Selection Method
Source: J Med Internet Res. 2019 Apr 2;21(4):e12437. doi: 10.2196/12437 (PMC6466481; doi:10.2196/12437)
Supplement: Multimedia Appendix 2 [file jmir_v21i4e12437_app2.pdf]

Appendix 2: Weighting of the 28 Features Based on IG.

Feature	IG	C	q	Accuracy (%)	Contribution	IG (0-1)	Contribution (0-1)	Weight
AGE	0.057	4	256	54.8	-	1.00	1.00	2.00
Apo-B	0.040	256	256	58.8	4.04	0.70	0.44	1.14
TBIL	0.029	256	128	60.4	1.52	0.52	0.26	0.78
Apo-A1	0.026	256	128	59.8	-0.51	0.45	0.12	0.58
Glu	0.024	256	128	61.0	1.14	0.42	0.24	0.65
DBIL	0.015	256	128	62.2	1.26	0.26	0.25	0.51
BMI	0.012	32	256	60.0	-2.27	0.21	0.00	0.21
CK-MB	0.009	8	256	59.7	-0.25	0.17	0.14	0.31
Gender	0.008	8	256	60.4	0.63	0.13	0.20	0.34
Height	0.007	256	2	72.5	12.12	0.12	1.00	1.12
ALT	0.006	128	1	71.5	-1.01	0.11	0.09	0.20
ALP	0.006	64	0.25	70.3	-1.14	0.11	0.08	0.18
AST	0.006	256	0.5	71.3	1.01	0.10	0.23	0.33
BUN	0.005	128	0.5	71.2	-0.13	0.08	0.15	0.23
PI	0.005	128	0.5	71.2	0.00	0.08	0.16	0.24
LDH	0.005	64	1	72.3	1.14	0.08	0.24	0.32
SCr	0.004	128	1	73.7	1.39	0.06	0.25	0.32
CK	0.003	256	1	73.1	-0.63	0.05	0.11	0.17
GGP	0.003	256	1	73.2	0.13	0.05	0.17	0.22
TP	0.002	16	2	73.2	0.00	0.04	0.16	0.20
TC	0.002	16	2	73.2	0.00	0.04	0.16	0.20
LDL	0.001	128	1	72.9	-0.38	0.02	0.13	0.16
Alb	0.001	128	1	74.1	1.26	0.02	0.25	0.27
UA	0.000	128	0.25	73.5	-0.63	0.00	0.11	0.12
HDL	0.000	128	0.25	73.6	0.13	0.00	0.17	0.17
α-HBD	0.000	128	1	73.2	-0.38	0.00	0.13	0.13
TG	0.000	128	1	73.0	-0.25	0.00	0.14	0.14
Ca	0.000	128	1	73.0	0.00	0.00	0.16	0.16
